# The Unusual Acid-Accumulating Behavior during Ripening of Cherimoya (*Annona cherimola* Mill.) is Linked to Changes in Transcription and Enzyme Activity Related to Citric and Malic Acid Metabolism

**DOI:** 10.3390/molecules21050398

**Published:** 2016-04-25

**Authors:** Mauricio González-Agüero, Luis Tejerina Pardo, María Sofía Zamudio, Carolina Contreras, Pedro Undurraga, Bruno G. Defilippi

**Affiliations:** 1 Instituto de Investigaciones Agropecuarias, INIA La Platina, Santa Rosa 11610, Santiago 8831314, Chile; sofizamudio@gmail.com (M.S.Z.); cdcontr1@uc.cl (C.C.); bdefilip@inia.cl (B.G.D.); 2 Facultad Ciencias Biológicas, Escuela de Ingeniería en Biotecnología, Universidad Andrés Bello, República 217, Santiago 8370146, Chile; ltejerinapardo@gmail.com; 3 Facultad de Agronomía, Pontificia Universidad Católica de Valparaíso, Calle San Francisco s/n, Casilla 4-D, Quillota 2260000, Chile; pundurra@ucv.cl

**Keywords:** *Annona cherimola*, organic acids, gene expression

## Abstract

Cherimoya (*Annona cherimola* Mill.) is a subtropical fruit characterized by a significant increase in organic acid levels during ripening, making it an interesting model for studying the relationship between acidity and fruit flavor. In this work, we focused on understanding the balance between the concentration of organic acids and the gene expression and activity of enzymes involved in the synthesis and degradation of these metabolites during the development and ripening of cherimoya cv. “Concha Lisa”. Our results showed an early accumulation of citric acid and other changes associated with the accumulation of transcripts encoding citrate catabolism enzymes. During ripening, a 2-fold increase in malic acid and a 6-fold increase in citric acid were detected. By comparing the contents of these compounds with gene expression and enzymatic activity levels, we determined that cytoplasmic NAD-dependent malate dehydrogenase (*cyNAD-MDH*) and mitochondrial citrate synthase (*mCS*) play important regulatory roles in the malic and citric acid biosynthetic pathways.

## 1. Introduction

During ripening, fruits are transformed into palatable consumer products, and consumer preference for a specific fruit or variety is determined by its organoleptic attributes. These attributes depend on many factors, such as the genotype, growing conditions, harvest time and storage conditions. Moreover, organoleptic qualities are related to many other characteristics such as firmness, color, aroma, sweetness and acidity, which are associated with specific metabolic pathways that are coordinated during fruit development and ripening [1]. Throughout this process, a series of changes in fruit composition occur, including the synthesis and degradation of pigments, volatile compound accumulation, and changes in the concentration of sugars and organic acids [2].

Flavor, one of the most important traits affecting fruit quality, is a complicated attribute that is mainly controlled by the level and ratio of different sugars and organic acids [3]. In fruits, these components are involved in both primary metabolism and in the biosynthesis of secondary metabolites, including amino acids, vitamins and aroma volatiles, which further influence quality [4]. Sugars and organic acids, the traits most commonly associated with fruit taste, are mainly measured as total soluble solids (TSS) and titratable acidity (TA), respectively [5], and the relationship between TSS and TA plays an important role in consumer acceptance of several species, such as citrus [6], table grapes [7] and cherries [8].

In fruit species such as apples, grapes and tomatoes, acidity has long been considered one of the most important quality attributes [9,10]. Several organic acids contribute to the acidity levels in fruit, though citric (or its conjugate base citrate) and malic acids (or malate) are the major contributors [11]. Organic acids accumulate during fruit development and are consumed as respiratory substrates in glycolysis and the tricarboxylic acid (TCA) cycle; these acids also serve as gluconeogenesis substrates during fruit ripening [10].

Inside the cell, malic acid is formed from phosphoenolpyruvate (PEP) through the activities of phosphoenolpyruvate carboxylase (*PEPC*) and malate dehydrogenase (MDH) [9], and cytosolic malate is transported to and sequestered in the vacuole [12]. Malate can be degraded by the reversible reaction of MDH and NADP-malic enzyme (*NADP-ME*).

Citric acid synthesis is thought to occur in the mitochondria via the TCA cycle. Mitochondrial citrate synthase (*mCS*) is proposed to catalyze the controlling step of citrate synthesis, with aconitase (*ACON*) and NAD-dependent isocitrate dehydrogenase (NAD-IDH) controlling citrate degradation [10]. Although *mCS* activity has been positively correlated with citrate accumulation in citrus and strawberries [13,14], the lower activity of *ACON*, which catalyzes the conversion of citrate into isocitrate, could be responsible for the increase in citrate concentration during fruit growth in sour lemons [15]. NAD-IDH is present in mitochondria but rarely identified in fruits, and no links with citrate accumulation have been found [16]. Therefore, the molecular mechanism responsible for citrate degradation remains largely unknown.

In general, fruit ripening is accompanied by organic acid degradation and therefore a decrease in acidity, as has been described in such fruits such as pears [16], peaches [17], and apples [18]. However, some fruit species exhibit different acidity patterns during ripening. For example, an increase in TA has been described during ripening in “mangaba” (*Hancornia speciosa*), a tropical plant native of Brazil, “soursop” (*Annona muricata*) and cherimoya [19,20,21]. In particular, the rise in acidity in cherimoya is attributed to the production of organic acids during ripening [22,23] and to higher malic acid accumulation caused by the action of MDH as the fruit ripens [24]. However, little is known about the reason for this particular trend in cherimoya and the pathways responsible for the synthesis, regulation and accumulation of organic acids during development and ripening.

In this study, we focused on understanding the balance between organic acid levels during cherimoya development and ripening and enzymatic activity/gene expression related to the synthesis and degradation of these metabolites. For this purpose, cherimoya cv. “Concha Lisa” was collected at different growth stages, and mature samples were ripened at 20 °C. Standard fruit quality parameters, the organic acid content, enzymatic activity and the expression of several key genes encoding putative enzymes related to citric and malic acid metabolism were evaluated. Our results are expected to provide baseline information for elucidating the mechanisms involved in organic acid metabolism in ripening *A. cherimola* fruit.

## 2. Results and Discussion

### 2.1. Changes in Organic Acid Content during Growth of Cherimoya

Fruits of cherimoya cv. “Concha Lisa” at nine different growth stages were collected between January and November 2013 (Table 1); the fruits were classified by phenological stage using the *Biologische Bundesanstalt*, *Bundessortenamt und Chemische Industrie* (BBCH) scale, according to Cautín and Agustí [25]. For example, the initial state collected (S1) corresponds to growth stage 71 at the beginning of ovary growth, a time when the green ovary is surrounded by the dying petal crown. An intermediate stage of development (S5) corresponds to stage 75, at which the fruit has reached half of its final size [25]. Other developmental stages and their equivalence in the BBCH-scale are described in Table 1. As development progresses, both fruit weight and diameter (data not shown) exhibit a double sigmoid curve with a short latency period (stage II), which is typical growth for this fruit during a season with predominantly low temperatures [26]. Although a series of studies have examined the composition of organic acids during cherimoya ripening [21,23,24,27], there is no evidence to date on what occurs during the development of this fruit.

Our analysis showed the predominance of citric acid in the early stages of development, decreasing to very low levels (0.20 mg/g of fresh weight [FW]) when the fruits reached the commercial harvest stage. In contrast, the malic acid levels remained constant throughout the development, becoming the predominant acid at nearly 64% of the total acids analyzed at harvest. This is consistent with the reports of Paull *et al.* [20] and Alique *et al.* [27], who found that malic acid is the major organic acid contributing to the increase in TA in soursop and cherimoya, respectively. Levels of both malate and citrate were also highly correlated with many important regulators of ripening in an independent study focused on early tomato fruit development [28]. As opposed to the observations for cherimoya, malic acid increases in other fruits such as grape (*Vitis vinifera*) at earlier stages and then decreases during ripening [29]. In early stages of development, malic acid primarily accumulates through the metabolism of sugars, whereas malate acts as a vital source of carbon for different pathways, mainly the TCA cycle, respiration, gluconeogenesis and secondary compound production, during ripening [30].

With regard to the other acids analyzed, such as tartaric, succinic and ascorbic, the general trend was a decrease in content as the fruits developed, showing no major changes from 17 weeks after blooming (WAB) until harvest. It is noteworthy that the high levels of succinic acid in the early stages of development decreased to below 0.4 mg/g FW at harvest. Similar results were reported by Fortes *et al.* [31] in grapes, in which succinic acid may serve as a substrate for the synthesis of primary sugars and other metabolites. Tartaric acid accumulated during the early fruit stages and remained unchanged for most of cherimoya development. Other authors have reported this pattern, whereby tartaric acid remains low during ripening because it is not metabolized in the fruit [32].

### 2.2. Sequence Analysis of Organic Acid-Related Genes from Cherimoya

Full-length cDNAs encoding cytoplasmic NAD-dependent malate dehydrogenase (*cyNAD-MDH*), NADP-dependent malic enzyme (*NADP-ME*), NAD-dependent malic enzyme (*NAD-ME*), ATP citrate synthase (*ATP-CS*) also called ATP-citrate lyase, NADP-dependent isocitrate dehydrogenase (*NADP-IDH*) and mitochondrial citrate synthase (*mCS*) were identified, as were partial-length transcripts sequences encoding phosphoenol pyruvate carboxylase (*PEPC*), aconitate hydratase 1 (*ACON*). BLASTp was used to confirm homology, and the protein products were designated *Ac-cyNAD-MDH* (332 amino acids—aa), *AcNADP-ME* (587 aa), *AcNAD-ME* (606 aa), *AcATP-CS* (423 aa), *AcNADP-IDH* (416 aa), *Ac-mCS* (473 aa), *AcPEPC* (164 aa), and *AcACON* (473 aa). The data provided in Table 2 indicate high identity of cherimoya malic and citric acid-related genes with orthologous genes.

### 2.3. Expression of Citric and Malic Acid-Related Genes during A. cherimola Fruit Growth

**Figure 1:**
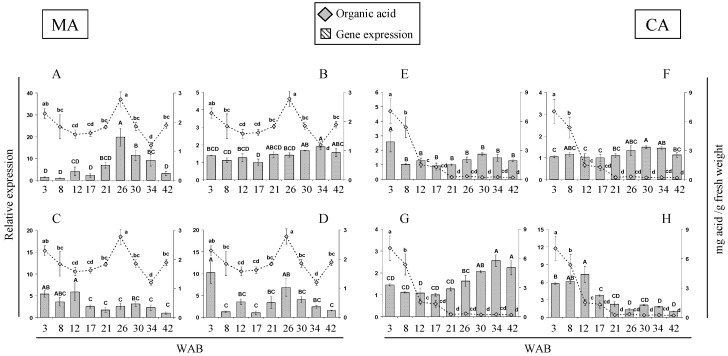
Expression profiles of putative organic acid-related genes during the growth of cherimoya cv. “Concha Lisa”. Quantitative real-time PCR assays were performed in duplicate on samples from 3 to 42 weeks after blooming (WAB). The genes analyzed related to malic acid (MA) were, (**A**) phosphoenol pyruvate carboxylase, *AcPEPC*; (**B**) cytoplasmic NAD-dependent malate dehydrogenase, *Ac-cyNAD-MDH*; (**C**) NADP-dependent malic enzyme, *AcNADP-ME*; and (**D**) NAD-dependent malic enzyme, *AcNAD-ME*. The genes analyzed related to citric acid (CA) were; (**E**) citrate synthase, *Ac-mCS*; (**F**) aconitate hydratase 1, *AcACON*; (**G**) NADP-dependent isocitrate dehydrogenase, *AcNADP-IDH*; and (**H**) ATP citrate synthase, *AcATP-CS*. Expression was normalized to a reference gene (*AcUbiq*, GenBank FJ664263) and the value is expressed against the lowest value of relative abundance. The relative expression of each gene (bars) was compared with the concentration of the respective acid (gray rhombus). Different letters (capitals for expression and lower cases for activity) between each time point represent significant differences at *p* ≤ *0.05* by the LSD test.

The expression of four genes related to malic acid metabolism and four citric acid-related genes was analyzed by real-time quantitative PCR (qPCR) in developing fruits at nine stages (Figure 1). Expression patterns for these organic acid-related genes or the activities of key enzymes differ among species, such as peach [17], strawberry [14], apple [15], pear [16], and melon [34]. Although no major changes in malic acid were found during cherimoya fruit development, the expression patterns of genes related to its synthesis and degradation do not necessarily correlate with the timing of the observed changes in organic acid content (Figure 1). *AcPEPC*, which codes for the enzyme responsible for producing oxaloacetate (OAA) at the beginning of the malate synthetic pathway, showed the highest expression at 26 WAB and then declined (Figure 1A). This expression pattern correlates with the malate concentration. Another gene involved in the synthesis of malate, *Ac-cyNAD-MDH*, did not exhibit significant changes in expression, except for a slight increase in the last stages of fruit development (Figure 1B). The profiles of the *AcPEPC* and *Ac-cyNAD-MDH* genes might explain the pattern of a low malate exchange rate; similarly, decreases in the expression of genes, *AcNADP-ME* (Figure 1C) and *AcNAD-ME* (Figure 1D), responsible for the degradation of this metabolite may also explain this behavior during cherimoya development.

Regarding citric acid metabolism, the expression patterns of *Ac-mCS* (Figure 1E), which is implicated in citrate synthesis, *AcACON* (Figure 1F) and *AcNADP-IDH* (Figure 1G), both related to citrate degradation, may account for the high accumulation of this acid at the initial stages and their rapid decline as cherimoya development progresses. Interestingly, a relationship between *Ac-mCS* and the citric acid pattern was found in cherimoya; this is different from that observed by Iannetta *et al.* [14] in strawberry, where *Fa-mCS1* expression did not correlate with either enzyme activity or citric acid accumulation. In the case of *AcATP-CS* (Figure 1H), a gene possibly related to citrate degradation, the expression pattern showed no relationship with the accumulation of this organic acid.

### 2.4. Changes In Quality Parameters and Organic Acid Patterns during Cherimoya cv. “Concha Lisa” Ripening

As expected, firmness was very high at harvest, reaching values of 18 kgf and remaining constant until day 2, at which a rapid softening was observed at 20 °C (Table 3). This change in pulp firmness coincided with a significant increase in ethylene production after 5 days, with a peak observed at 8 days at 20 °C. Importantly, we observed the particular behavior of cherimoya as a climacteric fruit that shows a double peak in respiratory rate [27,35]. Moreover, an increase in TSS and TA was observed during cherimoya ripening (Table 3), similar to the results reported by Manríquez *et al.* [23]. The TA increases coincidently with the respiratory rate, and after 5 days at 20 °C the TA shows an increase (2.4 fold) immediately after the first CO_2_ peak. This result is similar to that reported in soursop, with the authors suggesting that the increase might be a consequence of the glycolysis induced by harvest, with intense glucose oxidation and starch hydrolysis [36].

In relation to the organic acid content, our analysis revealed almost no changes in ascorbic, tartaric and succinic acid levels during cherimoya ripening (Table 3); this was similar to what was found by Manríquez *et al.* [23] in “Concha Lisa” and “Bronceada” (another cherimoya cv.), where the tartaric acid concentration was very low from harvest to the last stages of ripening. In contrast, malic and citric acids were predominant and increased significantly as the fruits ripened; indeed, the ripening of “Concha Lisa” cherimoya fruit was accompanied by a 2-fold increase in malic acid and a 6-fold increase in citric acid. Both acids peaked at 5 days after harvest, declining thereafter and remaining constant (Table 3). Interestingly, the increase in malic and citric acids at 20 °C parallels the increase in TA, suggesting that both acids may be responsible for the changes in TA during cherimoya ripening.

### 2.5. Gene Expression and Activity Analysis of Key Enzymes Related to Malic and Citric Metabolism during Cherimoya Ripening

**Figure 2:**
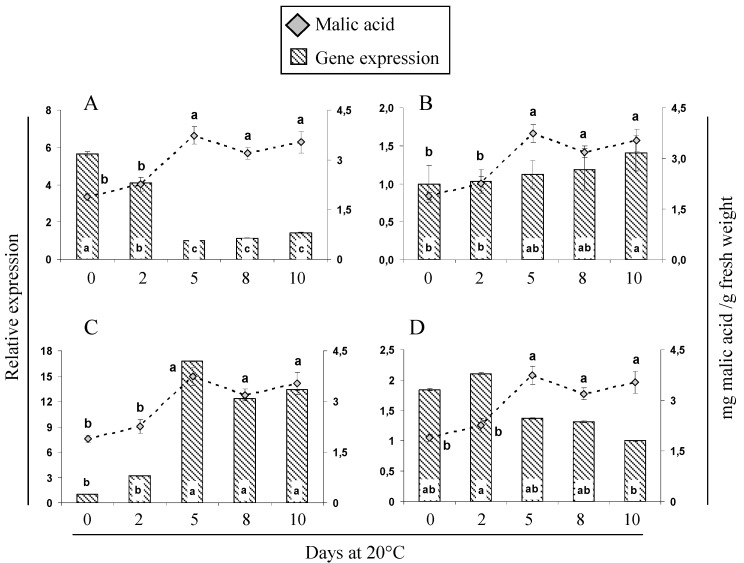
Expression profiles for four genes related to malic acid metabolism during ripening of “Concha Lisa” cherimoyas at 20 °C. The following genes were analyzed by qPCR: (**A**) phosphoenol pyruvate carboxylase, *AcPEPC*; (**B**) cytoplasmic NAD-dependent malate dehydrogenase, *Ac-cyNAD-MDH*; (**C**) NADP-dependent malic enzyme, *AcNADP-ME*; and (**D**) NAD-dependent malic enzyme, *AcNAD-ME*. Expression was normalized to a reference gene *AcUbiq* (GenBank FJ664263) and the value is expressed against the lowest value of relative abundance. The expression of each gene (bars) was compared with the concentration of malic acid (gray rhombus). Different letters between each sample point represent significant differences at *p* ≤ 0.05 by the LSD test.

The expression of eight genes and the activity of three enzymes were analyzed and compared to the increase in the respective organic acids during cherimoya ripening. Four genes related to malic acid metabolism (Figure 2) were analyzed by qPCR. One of them, *AcPEPC* (Figure 2A) showed high expression in the first days of ripening and then decreased; however, this could not explain the increase in malic acid detected during cherimoya ripening. Rises in malic acid concentration increases the availability of OAA during the first days of ripening, and this OAA is subsequently metabolized in other fruit species such as melon [34] and loquat [37]. *PEPC* activity increased during the early stages and then decreased, revealing a relationship between *PEPC* activity and organic acid concentration [34].

**Figure 3:**
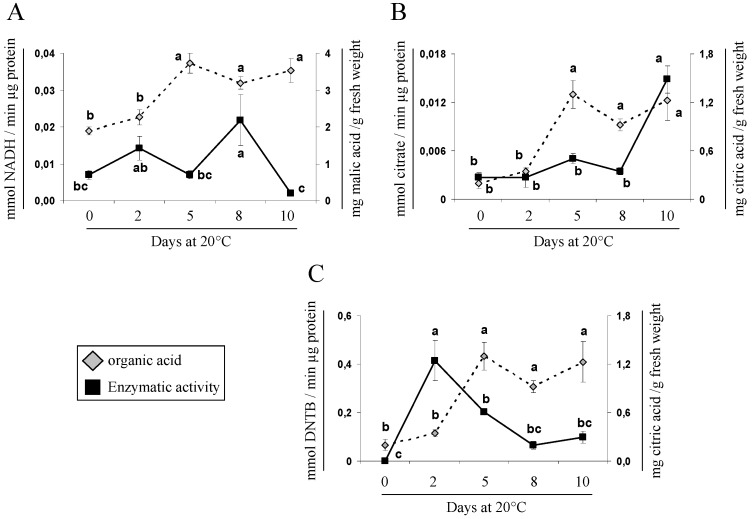
Activity profiles of organic acid-related enzymes during “Concha Lisa” cherimoya ripening. Assays were performed in duplicate using samples of 4 fruits per time point during ripening at 20 °C. The following enzymes were analyzed: (**A**) NAD-dependent malate dehydrogenase, NAD-MDH; (**B**) citrate synthase, CS; and (**C**) aconitate hydratase, *ACON*. In each panel, enzymatic activity (black rectangles) is related to and compared with the concentration of the respective organic acid (gray rhombus). Different letters between each sample point represent significant differences at *p ≤* 0.05 by the LSD test.

In the case of *Ac-cyNAD-MDH* (Figure 2B), which is responsible for the synthesis of malate from OAA in the cytoplasm, a slight increase in expression was found when the fruits ripened (10 days at 20 °C), and this could be related to the increase in malic acid concentration. Likewise, an increase in the NAD-MDH activity (8 days at 20 °C) was observed (Figure 3A). NAD-MDH activity represents a combination of cytosolic and mitochondrial activities; therefore it is not uncommon to find some divergence between activity data and the expression of this cytoplasmic gene. While, there is no temporal coincidence between the perceived rise on *Ac-cyNAD-MDH* expression and NAD-MDH activity; both, gene expression and protein activity increase and may be related with the high accumulation of malic acid. These results are consistent with and support the observations of Muñoz *et al.* [24], who suggested that the *in vitro* properties of these enzymes and the fact that the activity of NAD-MDH is higher than the activity of *NADP-ME* account for the particularly high titratable acidity of “Fino de Jete” cherimoyas during ripening.

Although we did not measure the activity of *NADP-ME*, the enzyme responsible for malic acid degradation, we did analyze expression of the *AcNADP-ME* gene (Figure 2C). We found increased ME transcript abundance, which was not correlated with the malate behavior. Both, the decrease in expression of *Ac-PEPC* and the increase in *AcNADP-ME*, may suggest that the biosynthesis of malate that normally occurs via *PEPC* and NAD-MDH, switches to pyruvate kinase and *NADP-ME* activities during ripening, similar to what has been found in other fruits, including non-climacteric fruits such as grapes [9]. Moreover, the *AcNAD-ME* gene, encoding the enzyme responsible for the conversion of malic acid to pyruvate, showed a decrease in expression at the end of ripening (Figure 2D).

Chen *et al.* [37] reported that ME may play a significant role in decreasing malic acid concentrations during the ripening of loquats, which is different from the observations of Saradhuldhat and Paull [38], who described no correlation with malic acid in pineapples. These different results show how interesting it would to learn more about the activity of this enzyme and its intracellular environment in different species.

**Figure 4:**
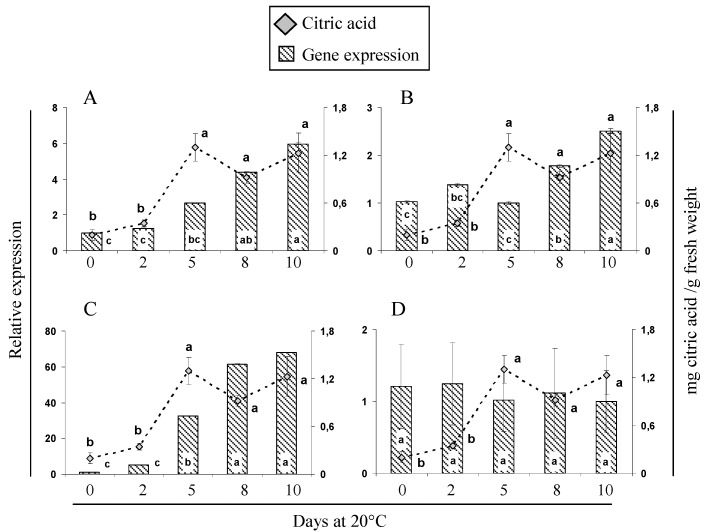
Expression profiles of putative genes related to the synthesis or degradation of citric acid during ripening of cherimoya cv. “Concha Lisa”. The following genes were analyzed by qPCR: (**A**) citrate synthase, *Ac-mCS*; (**B**) aconitate hydratase 1, *AcACON*; (**C**) ATP citrate synthase, *AcATP-CS*; and (**D**) NADP-dependent isocitrate dehydrogenase, *AcNADP-IDH*. Expression was normalized to the reference gene *AcUbiq* (GenBank FJ664263) and the value is expressed against the lowest value of relative abundance. In each panel, gene expression (bars) was compared with the concentration of citric acid (gray rhombus). Different letters between each time point represent significant differences at *p* ≤ 0.05 by the LSD test.

With regard to citric acid metabolism, the first step in the TCA cycle is catalyzed by *mCS*. Our results showed that the increase in citric acid concentration was accompanied with enhanced CS activity (Figure 3B) and *Ac-mCS* gene expression (Figure 4A). These findings suggest that *mCS* may play a significant role in citric acid biosynthesis during ripening of “Concha Lisa” cherimoyas, similar to what has been reported in melons by Tang *et al.* [34]. Nonetheless, as citric acid is degraded by *ACON* in the TCA cycle, the citric acid concentration is expected to increase as *ACON* activity decreases.

Figure 4B shows that the citric acid concentration in cherimoya increased linearly, with a significant rise in *AcACON* expression, at the final stages of ripening. However, *ACON* activity increased rapidly after harvest but declined after 2 days at 20 °C (Figure 3C), consistent with previous findings in lemon, whereby reductions in *ACON* activity were found to play a role in citric acid accumulation [15,39]. Morgan *et al.* [39] report an increase in citrate proportional to the decrease in aconitase during ripening of transgenic and introgression lines of tomatoes, suggesting that aconitase activity is a major determinant, which affects not only the citrate levels, but also led to a substantial increase in malate and a decrease in succinate and fumarate concentrations on ripe fruits. Otherwise, similarly to the observations for *AcNADP-ME* in malate metabolism, expression of the *AcATP-CS* gene, which is also involved in the degradation of citric acid, surprisingly increased to approximately 60 times higher than the minimum value expressed during cherimoya ripening (Figure 4C); this was opposite to the citric acid content measured. However, this significant increase in *AcATP-CS* expression could be related to an increase in activity of this enzyme, which has been reported during ripening of mango fruit [40]. The increase of *ATP-CS* activity could switch the citrate metabolism away from the TCA cycle towards the supply of acetyl-CoA and OAA for other metabolically active pathways (e.g., flavonoids and isoprenoids) during fruit ripening [2]. Importantly, the malate and citrate metabolism are intimately connected, and the net synthesis of malate by *ATP-CS* has yet to be demonstrated in fruits [9], but to the best of our knowledge, this is the first report about it fact. Finally, *AcNADP-IDH* (Figure 4D), which is indirectly involved in the degradation of citrate, remained unchanged during ripening. Previous studies in peach [17] and melon [34] have shown that the expression patterns of genes involved in organic acid metabolism are not necessarily correlated with changes in organic acid content.

## 3. Materials and Methods

### 3.1. Plant Materials

Samples of developing cherimoya cv. “Concha Lisa” fruits were obtained from trees located at the La Palma experimental station at the Pontificia Universidad Católica de Valparaíso, Quillota (Chile), latitude −32.898574°, longitude −71.210712° and elevation 200 m. Samples were collected at nine stages of development between January and November 2013, and the phenological stage was classified according to Cautín and Agustí [25] using the BBCH-scale. The samples were transported to the Postharvest Laboratory facility at INIA to record the total fruit weight and equatorial diameter and were subsequently stored at −80 °C until use. Harvest-mature (November) samples were collected considering ground color as a harvesting index and then ripened at 20 °C for up to 10 days until the ready-to-eat stage was reached.

### 3.2. Fruit Parameters

Quality parameters were determined for each sample point during ripening from six fruits as biological replicates, and the results were expressed as the mean ± standard error (SE). Ethylene production and the respiration rate were determined using intact fruits with a static system. Briefly, fruits from each ripening stage were weighed and placed in 1.56-L jars, which were sealed and kept at 20 °C for 30 min prior to measurements. The concentrations of carbon dioxide (mL CO_2_ kg^−1^·h^−1^) and ethylene (µL C_2_H_4_ kg^−1^·h^−1^) in the jar headspace were then determined using a gas analyzer (PBI-Dansensor Checkmate 9900, Ringsted, Denmark) and a gas chromatograph (Shimadzu 8A, Tokyo, Japan) equipped with a flame ionization detector (FID). Fruit firmness was assessed by two measurements performed on opposite sheets of peeled fruit using a penetrometer (Effegi, Milan, Italy) equipped with a 4-mm (at harvest) or 8-mm (when fruits were nearly ripe) plunger; the values are expressed in kgf. Each fruit was subsequently halved, and one half was immediately frozen in liquid nitrogen and stored at −80 °C until further analysis. From the other half, a sample of 10 g was homogenized in a mortar, and the juice was analyzed for TSS and TA. TSS was measured using a temperature-compensated refractometer (ATC-1e, Atago, Tokyo, Japan), and the value is expressed as a percentage. TA measurements were performed by titration with 0.2 N NaOH until pH 8.2 was reached; the values were expressed as a percentage of malic acid.

### 3.3. Organic Acid Extraction and Measurement

Samples from each developmental stage and ripening point were prepared from 5 g of frozen homogeneous tissue sample from each fruit; six replicates at each sampling time were considered. Organic acids were extracted and analyzed by HPLC according to the methodology described by Manríquez *et al.* [23]. Organic acids were analyzed in a chromatography system with an L-4250A UV-VIS ultraviolet detector (Merck-Hitachi, Tokyo, Japan) for absorbance measurement at 195 nm with a D-6000 interface (Merck-Hitachi). Concentrations of tartaric, malic, ascorbic, citric, and succinic acid were determined according to a standard curve; the values are expressed as mg**·**g^−1^ fresh weight (FW) of fruit. All standards were obtained from Sigma-Aldrich (St. Louis, MO, USA).

### 3.4. Crude Protein Extraction and Enzyme Assays

Total protein from each 3 g sample of homogenized tissue was extracted using 5 mL of grinding buffer (0.2 M Tris-HCL pH 8.2, 0.6 M sucrose and 10 mM isoascorbic acid); the mixture was centrifuged for 5 min at 4 °C at 4000× *g*, and the supernatant was centrifuged again (at 4000× *g*) for 10 min at 4 °C. The supernatant (4 mL) was mixed with 1 mL extraction buffer (0.2 M Tris-HCL pH 8.2, 10 mM isoascorbic acid, Triton 0.1% *v*/*v*), and 2 mL (tube A) was centrifuged at 15,000× *g* for 15 min at 4 °C; the supernatant was discarded, and the precipitate was resuspended in another 2 mL of extraction buffer. The volume of this fraction (3 mL) was brought to 6 mL with extraction buffer, another 2 mL aliquot was taken (tube B), and the remaining 4 mL (tube C) was dialyzed for 10 h against 800 mL of buffer. The protein concentration in the crude extract was determined by the method of Bradford [41] using the Bio-Rad protein assay reagent (Bio-Rad, Hercules, CA, USA) and bovine serum albumin as the standard. Extracts from tubes A, B and C were used for activity measurements with a Jenway 6715 UV/visible spectrophotometer (Bibby Scientific Limited, Staffordshire, UK). The reaction mixtures used for each enzyme according to Tang *et al.* [34] are given below. Each reaction was performed in a final volume of 500 µL, incubated at room temperature for 5 min, and started by the addition of the enzyme extract followed by immediate measurement after mixing.

- Aconitase (*ACON*): 40 mM Tris-HCl (pH 7.5), 0.1 M NaCl and 200 µM *cis*-aconitate, with 100 µL from tube A; measurement at 240 nm.
- Malate dehydrogenase (MDH): 40 mM Tris-HCl (pH 8.2), 2 mM MgCl_2_, 10 mM KHCO_3_, 0.5 mM GSH, 2 mM oxaloacetate and 150 µM NADH, with 100 µL from tube B; measurement at 340 nm.
- Citrate synthase (CS): 40 mM Tris-HCl (pH 9), 40 µM DTNB, 40 µM acetyl-CoA and 4 mM oxaloacetate, with 100 µL from tube C; measurement at 412 nm.

### 3.5. RNA Extraction and cDNA Synthesis

Total RNA from 2–4 g of frozen tissue from each development and ripening stage was extracted using the modified hot borate method [42]. The quantity of RNA was assessed using a Qubit^®^ 2.0 fluorometer (Invitrogen™, Eugene, OR, USA); the quality was assessed by measuring A260/280 and 260/230 ratios and by electrophoresis through a 1.5% formaldehyde-agarose gel. First-strand cDNA was obtained by reverse transcription reactions using 2 µg of DNase I-treated total RNA (Fermentas, Thermo Fisher Scientific Inc., Waltham, MA, USA) as the template, MMLV-RT reverse transcriptase (Promega, Madison, WI, USA) and oligo dT primers according to a standard procedure. The concentration of cDNA was assessed by measuring the absorbance at 260 nm. Each cDNA sample was diluted to 50 ng/μL prior to use in qPCR assays.

### 3.6. Isolation of Partial cDNAs for Organic Acid-Related Genes

To amplify cDNAs encoding isocitrate dehydrogenase (*NADP-IDH*, EC 1.1.1.42), aconitate hydratase 1 (*ACON*, EC 4.2.1.3), NADP-dependent malic enzyme (*NADP-ME*, EC 1.1.1.40), NAD-dependent malic enzyme (*NAD-ME*, EC 1.1.1.39), phosphoenolpyruvate carboxylase (*PEPC*, EC 4.1.1.31), mitochondrial citrate synthase (*mCS*, EC 2.3.3.1), ATP-citrate synthase (*ATP-CS*, EC 2.3.3.8) and cytosolic NAD-malate dehydrogenase (*cyNAD-MDH*, EC 1.1.1.37), heterologous or degenerate primers were designed according to conserved regions in the corresponding protein in other plant species; alternatively, those cited by Yang *et al.* [43], Moing *et al.* [44] or Iannetta *et al.* [14] were used. The sequences of the forward and reverse primers used are given in Table 4. In general, the PCR programs involved an initial denaturation step at 94 °C for 1 min, followed by 30–35 cycles of 94 °C for 30 s, 50–60 °C (depending upon the primers) for 30 s, and 72 °C for 60 s, and a final extension step at 72 °C for 10 min. All PCR reactions were performed in a MyCycler thermal cycler (Bio-Rad). The PCR products were verified by electrophoresis through a 1.5% (*w*/*v*) agarose gel containing ethidium bromide and then cloned into the pGEM-T Easy vector (Promega) followed by sequencing (Macrogen Corp., Seoul, Korea). All primary sequences were compared to sequences from National Center for Biotechnology Information (NCBI) using BLAST alignment programs [33].

### 3.7. Cloning of Full-Length Annona Cherimola Organic Acid-Related Genes

To obtain the full-length cDNAs of organic acid-related genes, *A. cherimola*-specific primers (Table 4) were designed using Primer Premier 5.0 software (Premier Biosoft International, Palo Alto, CA, USA). RACE-PCR assays were performed using the adaptors, primers, enzymes, and procedures of the GeneRacer kit (Invitrogen, Breda, The Netherlands). Amplified 5′ and 3′ RACE fragments were analyzed by agarose gel electrophoresis, and selected DNA fragments were cloned into pGEM T-Easy (Promega) according to the manufacturer’s recommendations. Both strands were sequenced and compared using BLAST alignment programs. The nucleotide sequence of each new A. cherimola organic acid-related gene was translated, and open reading frames (ORFs) were identified using ORF Finder [46] and Swiss-Model Tools [47]. The subcellular localization of predicted cytoplasmic and mitochondrial proteins, as based on the identification of a transit peptide, was analyzed using TargetP software [48].

### 3.8. Real-Time Quantitative PCR Assays

The transcript abundance of the nine organic acid-related genes identified in this study was analyzed by qPCR with a LightCycler^®^ 96 Real-Time PCR System (Roche Diagnostics, Mannheim, Germany) using LC-FastStart DNA Master SYBR Green I to measure amplified RNA-derived DNA products and gene-specific primers (Table 4), similar to García-Rojas *et al.* [49]. Real-time quantitative PCR was performed on each of four biological samples in duplicate, and the gene expression values were normalized to the *AcUbiq* (GenBank FJ664263) [45].

### 3.9. Statistical Analyses

All data were subjected to statistical analyses of variance, and the means were separated by an LSD test at the 5% significance level using Statgraphics Centurion Plus 5 (Manugistics Inc., Rockville, MD, USA).

## 4. Conclusions

In this work we conducted a comprehensive investigation of organic acid biosynthetic pathways in cherimoya fruit. To the best of our knowledge, this is the first study to evaluate the expression of genes, enzyme activities, and metabolites related to organic acid pathways, which are possibly related to the particular behavior of titratable acidity during cherimoya ripening. A total of eight novel *A. cherimola* genes showing various levels of expression throughout cherimoya development and ripening were identified and characterized. In addition, citric and malic acids were demonstrated to be the most abundant organic acids in “Concha Lisa” cherimoya fruits. These results reveal an early accumulation of citric acid as well as other changes associated with the abundance of transcripts encoding malate and citrate catabolism enzymes at the beginning of ripening; however, this stage may not define the particular acidity behavior of cherimoya during ripening. The increase in malic acid at the last stage may be due to increased activity of NAD-MDH during ripening, an increase in the transcript levels of *Ac-cyNAD-MDH* and down-regulation of *AcNAD-ME*. Similarly, the greater amount of citric acid could be due to an increase in the activity of CS, up-regulation of the *Ac-mCS* gene, and a decrease in *ACON* activity throughout *A. cherimola* ripening. Finally, the objective of this research was to understand why this particular increase in acidity occurs during cherimoya ripening; thus, future experiments for characterizing in more detail the individual components of organic acid metabolism (e.g., *Ac-mCS*) and other factors related to the transport and accumulation of organic acids could help broaden our understanding of the molecular mechanisms involved in these biosynthetic pathways in *A. cherimola*.

## Figures and Tables

**Table 1:** Organic acid contents of cherimoya fruit at nine stages of development, collected during 42 weeks. Results are expressed as the mean ± standard error (SE) of four separate extractions and determinations.

Sample Point	Weeks after Blooming (WAB)	Phenological Stage ^1^	Fruit Weight (g)	Organic Acids (mg/g Fresh Weight)				
				Tartaric	Malic	Ascorbic	Citric	Succinic
**S1**	3	71	1.1 ± 0.1 *^,^^h^	1.12 ± 0.01 ^b^	2.29 ± 0.17 ^a,b^	0.14 ± 0.01 ^b^	7.05 ± 1.25 ^a^	2.97 ± 0.69 ^a^
**S2**	8	72	3.4 ± 0.7 ^h^	1.34 ± 0.17 ^a^	1.84 ± 0.43 ^b,c^	0.18 ± 0.04 ^a^	5.37 ± 1.09 ^b^	0.62 ± 0.12 ^c^
**S3**	12	73	32.6 ± 4.0 ^g^	0.57 ± 0.06 ^c,d^	1.58 ± 0.15 ^c,d^	0.03 ± 0.01 ^c^	1.51 ± 0.31 ^c^	1.75 ± 0.29 ^b^
**S4**	17	74	70.5 ± 14.6 ^f^	0.40 ± 0.05 ^d,e^	1.62 ± 0.12 ^c,d^	0.03 ± 0.01 ^c^	0.97 ± 0.39 ^c,d^	0.62 ± 0.19 ^c^
**S5**	21	75	123.0 ± 14.4 ^e^	0.41 ± 0.03 ^c,d,e^	1.83 ± 0.07 ^b,c^	0.03 ± 0.00 ^c^	0.25 ± 0.04 ^d^	0.61 ± 0.14 ^c^
**S6**	26	76	186.8 ± 10.3 ^d^	0.61 ± 0.8 ^c^	2.78 ± 0.27 ^a^	0.05 ± 0.01 ^c^	0.29 ± 0.03 ^c,d^	0.85 ± 0.06 ^c^
**S7**	30	77	231.1 ± 15.7 ^c^	0.39 ± 0.7 ^d,e^	1.85 ± 0.17 ^b,c^	0.04 ± 0.01 ^c^	0.23 ± 0.02 ^d^	0.66 ± 0.03 ^c^
**S8**	34	78	328.5 ± 49.9 ^b^	0.25 ± 0.06 ^e^	1.20 ± 0.10 ^d^	0.04 ± 0.00 ^c^	0.20 ± 0.05 ^c,d^	0.55 ± 0.06 ^c^
**S9**	42	79	475.0 ± 74.4 ^a,b^	0.51 ± 0.15 ^c,d^	1.89 ± 0.11 ^b,c^	0.04 ± 0.00 ^c^	0.20 ± 0.07 ^d^	0.46 ± 0.05 ^c^

^1^ According to Cautín and Agustí [25], using the BBCH-scale. * In the same column, different letters indicated differences among development stages at *p* ≤ 0.05 by LSD test.

**Table 2:** Isolated genes related to organic acid metabolism in *Annona cherimola*.

Gene	GenBank Access	Length (aa)	Coding Enzyme and Type of Sequence Identified	Orthologous Sequence: Specie and GenBank Access ^1^	Identity (%)
*Ac-cyNAD-MDH*	KU524480	332	Cytosolic NAD-malate dehydrogenase, complete sequence	*Annona cherimola*—AHY99589	100
*AcNADP-ME*	KU524481	587	Putative NADP-dependent malic enzyme, complete sequence	*Theobroma cacao*—EOY15729	85
*AcNAD-ME*	KU524482	606	Putative NAD-dependent malic enzyme, complete sequence	*Theobroma cacao*—EOX96044	85
*AcATP-CS*	KU524483	423	Putative ATP-citrate synthase, complete sequence	*Ricinus communis*—XP_002512567	91
*AcNADP-IDH*	KU524484	416	Putative isocitrate dehydrogenase protein, complete sequence	*Prunus persica*—AAL11503	90
*Ac-mCS*	KU524485	473	Putative mitochondrial citrate synthase, complete sequence	*Cucumis sativus*—XP_004135902	88
*AcPEPC*	KU524486	164	Putative phosphoenolpyruvate carboxylase, partial sequence	*Elaeis guineensis*—AEQ94112	86
*AcACON*	KU524487	473	Putative aconitate hydratase 1, partial sequence	*Citrus clementina*—CBE71056	91

^1^ Access code according to BLASTp sequence alignment [33].

**Table 3:** Quality, physiological parameters and organic acid contents of cherimoya fruit during ripening at 20 °C. Results are expressed as the mean ± SE of a series of separate determinations and extractions. TSS: total soluble solids, and TA: titratable acidity.

		Days at 20 °C				
		0	2	5	8	10
**Quality and physiological parameters**	Firmness (kgf)	18.2 ± 2.2 *^,a^	18.1 ± 1.6 ^a^	1.6 ± 0.8 ^b^	0.5 ± 0.2 ^b^	0.6 ± 0.2 ^b^
	Respiratory rate (mL CO_2_ Kg^−1^·h^−1^)	17.3 ± 0.8 ^d^	67.6 ± 3.7 ^b^	54.7 ± 3.0 ^c^	121.7 ± 9.5 ^a^	138.8 ± 8.3 ^a^
	Ethylene rate (µl·Kg^−1^·h^−1^)	0.0 ± 0.0 ^c^	1.0 ± 0.3 ^c^	68.6 ± 13.3 ^b^	327.2 ± 38.0 ^a^	266.7 ± 49.6 ^a^
	TA (% malic acid)	0.09 ± 0.01 ^c^	0.10 ± 0.01 ^c^	0.22 ± 0.01 ^b^	0.30 ± 0.01 ^a^	0.25 ± 0.01 ^b^
	TSS (%)	6.8 ± 0.2 ^d^	10.2 ± 0.2 ^c^	15.5 ± 0.3 ^b^	16.2 ± 0.5 ^b^	18.2 ± 0.9 ^a^
**Organic acids (mg/g fresh weight)**	Tartaric	0.51 ± 0.15 ^a,b^	0.65 ± 0.07 ^a^	0.30 ± 0.04 ^b,c^	0.26 ± 0.05 ^c^	0.31 ± 0.06 ^b,c^
	Malic	1.89 ± 0.11 ^b^	2.26 ± 0.21 ^b^	3.73 ± 0.27 ^a^	3.19 ± 0.18 ^a^	3.53 ± 0.32 ^a^
	Ascorbic	0.04 ± 0.00 ^a^	0.04 ± 0.00 ^a^	0.05 ± 0.01 ^a^	0.05 ± 0.00 ^a^	0.05 ± 0.00 ^a^
	Citric	0.20 ± 0.07 ^b^	0.35 ± 0.04 ^b^	1.30 ± 0.17 ^a^	0.92 ± 0.08 ^a^	1.23 ± 0.25 ^a^
	Succinic	0.46 ± 0.05 ^a,b^	0.63 ± 0.06 ^a,b^	0.85 ± 0.27 ^a^	0.75 ± 0.20 ^a,b^	0.44 ± 0.07 ^b^

* In the same row, different letters indicated differences among sample points at *p* ≤ 0.05 by LSD test.

**Table 4:** Heterologous, degenerate and specific primers used for isolating cDNAs from cherimoya cv. “Concha Lisa”, RACE-PCR and qPCR assays.

Primer Name	Forward (*F*) and Reverse (*R*) Primers (5′→3′)	T_m_ (°C)	Reference
*PpNADP-IDH* heterologous primers (*Prunus persica*)	*F*: GGCCATGTACAACACTGATGAG	58	Yang *et al.* [43]
	*R*: CATTGTCATCCAACTTTGCCCTG		
*AcNADP-IDH*	*F*: TCCAAATGGAAGTCAAAGTTCGAG	64	N/A ^1^
	*R*: CCTTTCTGGTGAACACGGTAATG		
*CmACON* heterologous primers (*Citrus maxima*)	*F*: GAAGCAATCACCAAAGGGAATC	52	Yang *et al.* [43]
	*R*: TACTACGGTGAATTCGCTCAAAG		
*AcACON* RACE-PCR primers	*F*: ACCATGTCCCCTCCTGGCCC	64	N/A
	*R*: TCCACATTGGATTTCCTTTCGTG		
*AcACON* qPCR primers	*F*: GCAGGCACGGTTGACATTGATT	64	N/A
	*R*: GCCTGATGGCACGGATAATTGG		
*VvNADP-ME* heterologous primers (*Vitis vinifera*)	*F*: GGAGGAGTTCGTCCTTCAGCCTG	58	Yang *et al.* [43]
	*R*: CCTTTGAGTCCACAAGCCAAATC		
*AcNADP-ME* RACE-PCR primers	*F*: GCACAATCTCCGCCAATATGAAG	64	N/A
	*F*: AAGCCTCGCCAACGGTCGGT		
*AcNADP-ME* qPCR primers	*F*: AGAACGACTGGTCAGGAGTATG	64	N/A
	*R*: CCTCGCCTGCGCCAAGGAATA		
*VvNAD-ME* heterologous primers (*Vitis vinifera*)	*F*: TGATCTTGGAGTTCAGGGAATTGGA	58	Yang *et al.* [43]
	*R*: GCACTTCCTGCTCCAACTACAACTA		
*AcNAD-ME*	*F*: GCTGCTGGCATGAATCCACAAA	64	N/A ^1^
	*R*: GCGTGTAAAAACAGCTTCCATGA		
*PEPC* degenerate primers	*F*: CCITGGATHTTYGCITGGAC	54	Moing *et al.* [44]
	*R*: GCIGCDATICCYTTCATIG		
*AcPEPC* RACE-PCR primers	*F*: GAGACCCTGGTATTGCTGCTTT	64	N/A
	*R*: ACATTTTCTTGAGCATTTGGAGC		
*AcPEPC* qPCR primers	*F*: TGGACCCAGACCAGATTTCACC	66	N/A
	*R*: AATTGCTTCTCAGCCGCTCACC		
*mCS* degenerate primers	*F*: GGTTTRGCTGGRCCACTYC	46	Iannetta *et al.* [14]
	*R*: GRGCATCAACATTWGGC		
*Ac-mCS*	*F*: ATCAAGTCTGTTGTAGAAGAATGT	62	N/A ^1^
	*R*: CTCTGGCATGTGTATCGTGGA		
*AcATP-CS*	*F*: AAGTTCACCGTCCTCAACCCTA	62	González-Agüero *et al.* [45]
	*R*: GCATAACCCAAATCTCCAACAG		
*DEGcyNAD-MDH* degenerate primers	*F*: TTTCYATYTACAAGTCMCARGC	46	N/A ^2^
	*R*: CAGCTTWCGRGCCTTGATRATT		
*Ac-cyNAD-MDH*	*F*: AGAGTTTGCTCCATCCATTCCT	62	N/A ^1^
	*R*: CTCGTTGTTGGACGGTTGTAAT		
*AcUbiq* (Genbank FJ664263)	*F*: TCCTGCAGAATCAGTGGAGTC	64	González-Agüero *et al.* [45]
	*R*: AGGAACCAAATCCGCAAACAGC		

^1^ Specific primer for *A. cherimola* designed for RACE-PCR and also used for qPCR assays; ^2^ Degenerate primers designed using conserved domain by a ClustalW alignment from sequences of several plant NAD-MDHs.

## Abbreviations

The following abbreviations are used in this manuscript:

*cyNAD-MDH*: cytoplasmic NAD-dependent malate dehydrogenase
*mCS*: mitochondrial citrate synthase
TSS: total soluble solids
TA: titratable acidity
TCA: tricarboxylic acid cycle
PEP: phosphoenolpyruvate
*PEPC*: phosphoenolpyruvate carboxylase
*NADP-ME*: NADP-malic enzyme
*ACON*: aconitase
NAD-IDH: NAD-dependent isocitrate dehydrogenase
BBCH: *Biologische Bundesanstalt, Bundessortenamt und Chemische Industrie*
FW: fresh weight
WAB: weeks after blooming
*NAD-ME*: NAD-dependent malic enzyme
ATP-CS: ATP citrate synthase
aa: amino acids
qPCR: real-time quantitative PCR assays
OAA: oxaloacetate
SE: standard error
ORF: open reading frame
